# Genetic Labeling of Cells Allows Identification and Tracking of Transgenic Platelets in Mice

**DOI:** 10.3390/ijms22073710

**Published:** 2021-04-02

**Authors:** Irena Krüger, Friedrich Reusswig, Kim Jürgen Krott, Celina Fabienne Lersch, Martina Spelleken, Margitta Elvers

**Affiliations:** Department of Vascular and Endovascular Surgery, Experimental Vascular Medicine, Heinrich-Heine University Medical Center, 40225 Düsseldorf, Germany; Irena.Krueger@med.uni-duesseldorf.de (I.K.); FriedrichWilhelmBernd.Reusswig@med.uni-duesseldorf.de (F.R.); Kim-Juergen.Krott@med.uni-duesseldorf.de (K.J.K.); celer100@uni-duesseldorf.de (C.F.L.); Martina.Spelleken@med.uni-duesseldorf.de (M.S.)

**Keywords:** platelets, megakaryocytes, *loxP*, Cre recombinase, double fluorescent Cre reporter mouse, *PF4-Cre*

## Abstract

Background: The use of knock-out mouse models is crucial to understand platelet activation and aggregation. Methods: Analysis of the global double fluorescent Cre reporter mouse *mT/mG* that has been crossbred with the megakaryocyte/platelet specific *PF4-Cre* mouse. Results: Platelets show bright *mT* (*PF4-Cre* negative) and *mG* (*PF4-Cre* positive) fluorescence. However, a small proportion of leukocytes was positive for *mG* fluorescence in *PF4-Cre* positive mice. In *mT/mG;PF4-Cre* mice, platelets, and megakaryocytes can be tracked by their specific fluorescence in blood smear, hematopoietic organs and upon thrombus formation. No differences in platelet activation and thrombus formation was observed between *mT/mG;PF4-Cre* positive and negative mice. Furthermore, hemostasis and in vivo thrombus formation was comparable between genotypes as analyzed by intravital microscopy. Transplantation studies revealed that bone marrow of *mT/mG;PF4-Cre* mice can be transferred to *C57BL/6* mice. Conclusions: The *mT/mG Cre* reporter mouse is an appropriate model for real-time visualization of platelets, the analysis of cell morphology and the identification of non-recombined platelets. Thus, *mT/mG;PF4-Cre* mice are important for the analysis of platelet-specific knockout mice. However, a small proportion of leukocytes exhibit *mG* fluorescence. Therefore, the analysis of platelets beyond hemostasis and thrombosis should be critically evaluated when recombination of immune cells is increased.

## 1. Introduction

Platelets are major players in hemostasis and thrombosis. At sites of vessel injury, platelets adhere, become activated, and form a hemostatic plug to maintain the integrity of the vessel wall after vascular damage. Under pathological conditions, platelet activation leads to thrombosis at sites of atherosclerotic plaque rupture that obstructs the blood flow in the circulation. Arterial thrombi are platelet rich and induce myocardial infarction or stroke [1,2,3].

Injury of the vessel and extravasation of blood from the circulation into the surrounding tissue initiates events at the site of vessel damage and in blood to seal the injury. Circulating platelets are recruited to the site of injury by the interaction of platelet glycoprotein (GP) Ib and von Willebrand Factor (vWF) that is exposed on collagen of the extracellular matrix. This interaction is modulated by extracellular and intracellular platelet proteins such as reelin [4] and PLD1 [5] and captures platelets from the blood stream under high shear conditions, initiates low integrin α_IIb_β_3_ activation and allows the binding of the collagen receptor GPVI to collagen exposed at the injured vessel [6,7]. Activation of GPVI induces platelet activation characterized by Ca^2+^ mobilization, release of the second wave mediators adenosine diphosphate (ADP) and thromboxane, stable platelet adhesion and aggregation by integrin α_IIb_β_3_ and is reinforced by reelin that is released from platelets upon GPVI stimulation [7,8,9]. Concomitantly, blood coagulation is initiated by tissue factor leading to the generation of thrombin. Fibrinogen is cleaved by thrombin to fibrin monomers that polymerize and are cross-linked by the transglutaminase factor XIIIa to stabilize the growing thrombus [10]. The ability of platelets to provide a pro-coagulant surface to allow the assembly of the coagulation complex and the property of thrombin to activate platelets via protease-activated receptors indicates a crosstalk of platelet-mediated primary hemostasis and the coagulation cascade (secondary hemostasis).

In the last decades, new technologies including intravital video microscopy have been developed to analyze thrombus formation in a living mouse. Different mouse models have been established to explore the role of platelets, blood coagulation proteins, endothelium, and the vessel wall during thrombus formation in mice that offers a new understanding of the physiology and the pathology in complex biological systems. However, the investigation of single genes/proteins and their impact in platelet activation and thrombus formation is impeded by the fact that platelets are anucleate cells, which do not allow analysis by classical molecular biological means. In the last decades, animal models for in vivo generation of genetically modified platelets have been established to investigate these genes and their role in platelet activation and thrombus formation. The generation of (platelet-specific) knockout mice and the transplantation of genetically modified human or murine platelet progenitor cells in myelo-conditioned mice have been helpful in the understanding of new pathological pathways and the development of anti-thrombotic drugs [11]. The generation of platelet-specific knockout mice with the Cre-*loxP* method and a megakaryocyte/platelet-specific promoter has been shown to exhibit an advantage compared to constitutive knockout mice with a deletion of the target gene in all cells of the organism. This is due to the fact that the phenotype of these mice can be directed to megakaryocytes/platelets and is not affected by other cells. Furthermore, lethal phenotypes as observed, e.g., for talin, filamin A, or CLEC-2 are a limiting factor of the classical approach of whole organism knockout mice [12,13,14]. Thus, the use of inducible or cell type-specific promoters in combination with the Cre-*loxP* knockout technique allows spatiotemporal knockout or targeted expression in a particular cell type without the developmental effects or effects of gene knockdown in non-target cell types.

However, the generation of platelet-specific knockout mice is time-consuming and there are problems arising from these mouse models. A general problem is the existence of so-called endogenous pseudo-*loxP* sites, which can be targeted by Cre as well [15]. Furthermore, promoters that are only active during late stages of megakaryopoiesis such as the *platelet factor 4* (*PF4*) are not an appropriate model to study megakaryopoiesis [16,17]. The specificity of some of the promoters are not always fully restricted to one cell type as demonstrated by the erythrocyte/megakaryocyte-specific *GATA-1* promoter [18]. The promoter that drives the expression of the Cre recombinase has to be strong enough to gather complete DNA recombination important to generate a complete knockout of the target gene and to avoid the generation of mosaic animals. The *PF4* which is specific for the megakaryocytic lineage is nowadays used in many thrombosis studies with knockout mice and mice are born with a platelet-specific deletion of the target gene [19]. However, knockout efficiency of these conditional knockout mice is less than 95%, and thus wildtype platelets are still in the circulation of *PF4-Cre* mice and cannot be identified properly [19].

In this study, we were able to show that the global double fluorescent Cre reporter mouse *mT/mG* that has been crossbred with the *PF4-Cre* mouse is an efficient elegant model for megakaryocyte/platelet tracing, the analysis of cell morphology, transplantation studies and the differentiation of knockout versus wildtype platelets in these mice.

## 2. Results

### 2.1. Main Text

#### 2.1.1. Autofluorescence of Platelets in *mT/mG;PF4-Cre* transgenic Mice

**Figure 1:**
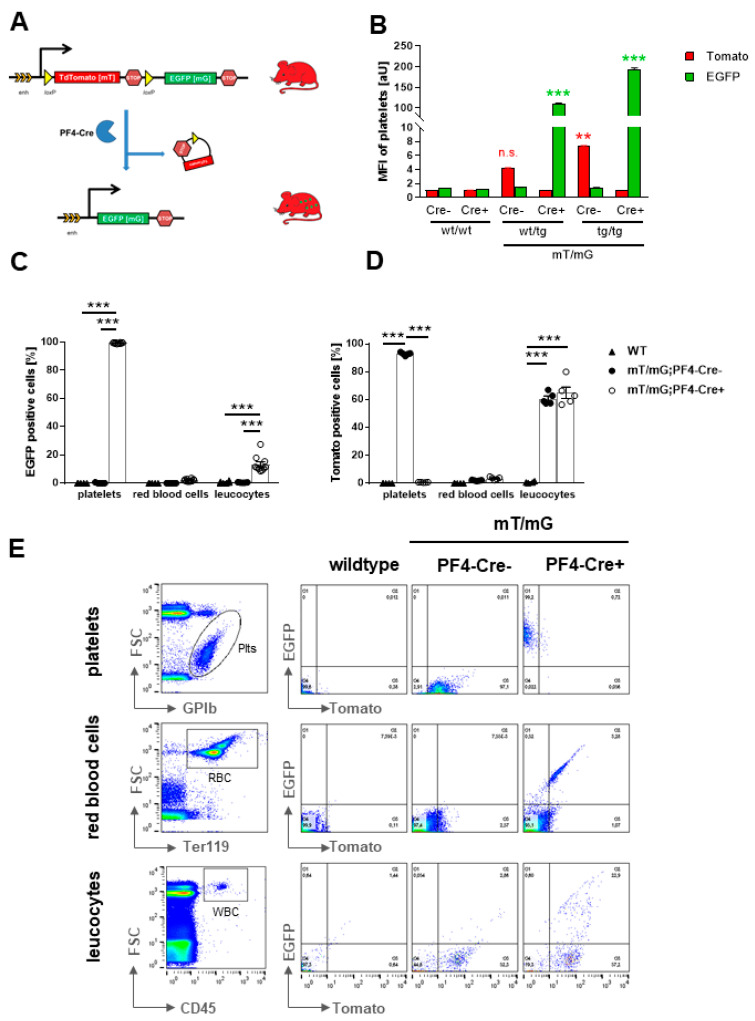
*mT* and *mG* fluorescence of blood cells in *mT*/*mG*; *PF4-Cre* reporter mice. (**A**) Schematic diagram of the genetic construct of *mT*/*mG*; *PF4-Cre* mice. The *mT*/*mG* construct consists of an enhancer (enh.) and a promoter (black arrow) region driving a loxP-flanked coding sequence of membrane-targeted tandem dimer Tomato (*mT*) resulting in tdTomato expression with membrane localization in every tissue. A stop codon inhibits the gene expression of membrane-targeted green fluorescent protein (*mG*). After Cre-mediated intrachromosomal recombination, the *mT* sequence is excised allowing the promoter/enhancer to drive the expression of *mG* in platelets and megakaryocytes. Yellow triangles represent loxP target sites for Cre-mediated recombination. (**B**) Mean fluorescence intensity (MFI) of *mT* and *mG* fluorescence in platelets regarding their specific side (SSC) and forward scatter (FSC) profile as determined by flow cytometry using whole blood of wildtype, heterozygous and homozygous *mT*/*mG PF4-Cre* negative and positive mice, n = 3–6. (**C**–**E**) Cell specific expression of *mT* or *mG* fluorescence was determined in platelets (DyLight649-labeled anti-mouse GPIbα), red blood cells (APC-labeled rat anti-mouse TER-119) and leucocytes (APC-labeled rat anti-mouse CD45). Fluorescence signals were detected in the FITC (*mG*) and PE (*mT*) channels. (**C**,**D**) MFI of *mG* (**C**) and *mT* (**D**) fluorescent cell populations in washed whole blood of WT and *mT*/*mG*;*PF4-Cre* negative and positive mice using flow cytometry, n = 5. (**E**) Representative dot blots showing the gating strategy for each cell population. The MFI of *mG* and *mT* fluorescence of different blood cells isolated from *mT*/*mG*;*PF4-Cre* mice. Statistical analysis was performed by Two-way Anova Sidak′s post hoc test (**A**,**B**) and Two-way Anova Tukey′s post hoc test (**C**,**D**). Bar graphs depict mean values ± SEM, * *p* < 0.05; ** *p* < 0.01 and *** *p* < 0.001.

The double-fluorescent Cre reporter mouse *mT/mG* has been crossbred with the megakaryocyte/platelet specific *PF4-Cre* mouse (Figure 1A). The *mT/mG* mouse expresses membrane-targeted tandem dimer Tomato (*mT*) prior to Cre-mediated excision and membrane-targeted green fluorescent protein (*mG*) after excision. As a result, *mT/mG;PF4-Cre* mice exhibit green fluorescent platelets in the circulation while platelets isolated from *PF4-Cre* negative mice show red fluorescence (Figure 1B). Fluorescence intensity of platelets can be observed already in heterozygous mice and is increased in the homozygous genetic background (Figure 1B). Fluorescence signals were detected in the fluorescein-5-isothiocyanate (FITC) (EGFP, *mG* fluorescence) and the phycoerythrin (PE) (Tomato, *mT* fluorescence) channels using *mT/mG;PF4-Cre* positive and negative heterozygous and homozygous mice. To confirm cell specificity of genetic labeling of blood cells in *mT/mG* mice, platelets, red blood cells and leukocytes were analyzed for their fluorescence characteristics by flow cytometry. The analysis of *mG* positive cells revealed no fluorescence of red blood cells in *PF4-Cre* negative and positive mice but strong *mG* fluorescence (99.46%) of platelets in *PF4-Cre* positive but not in *PF4-Cre* negative mice. However, we detected around 13% of *mG* positive leukocytes in *PF4-Cre* positive mice (Figure 1C). We next analyzed the number of *mT* positive blood cells and detected almost no fluorescence of red blood cells, neither in *PF4-Cre* negative nor positive mice (Figure 1D). As predicted, platelets from *PF4-Cre* negative mice exhibit strong *mT* fluorescence (92.92%) while platelets from *PF4-Cre* positive mice were negative for *mT* fluorescence. In contrast, strong *mT* fluorescence of leukocytes of both, *PF4-Cre* negative and positive mice, was detected (Figure 1D). Blood cells from *C57BL/6* mice were used as controls and show no *mG* and *mT* fluorescence as expected (Figure 1C,D). Fluorescence activated cell sorting of *mT* and *mG*-labeled blood cells was confirmed by their forward/sideward scatter profiles and cell type specific labeling of leukocytes (CD45), platelets (GPIb), and red blood cells (TER119) (Figure 1E). Taken together, nearly all leukocytes and platelets expressed a marker gene, either *mT* or *mG* while red blood cells exhibit only very low fluorescence signals. Although it is described that the *PF4-Cre* recombinase is restricted to the megakaryocyte/platelet lineage, a small population of leukocytes (13.39%) expressed *mG* while a large population of leukocytes (64.89%) express *mT*.

#### 2.1.2. Fluorescently labeled Platelets and Megakaryocytes in Blood Smear, Spleen, and Bone Marrow of Transgenic Mice

**Figure 2:**
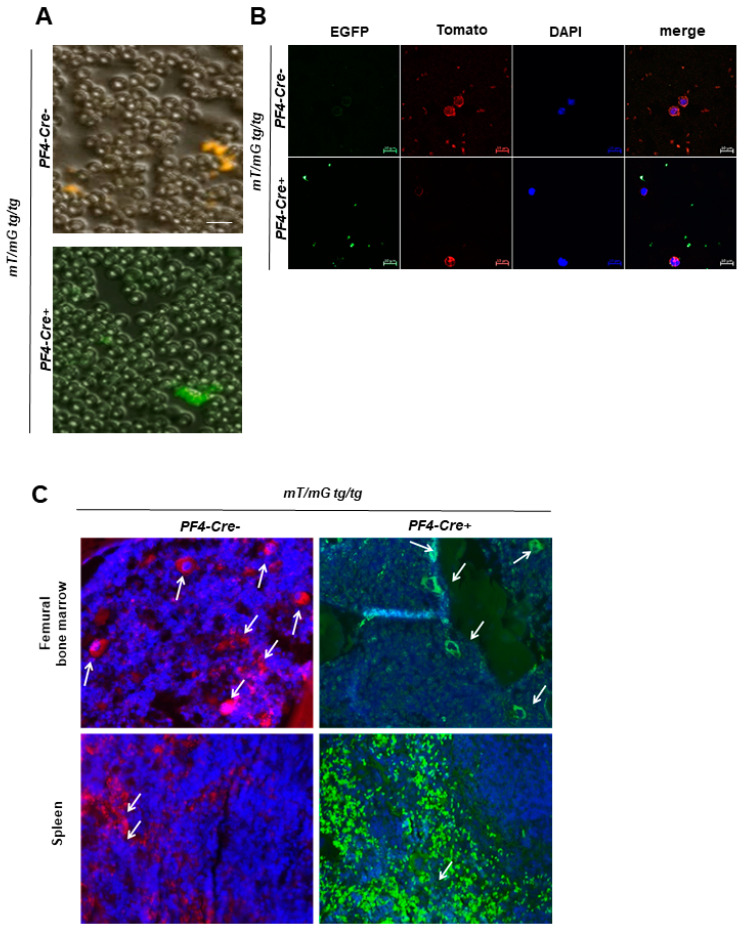
mT and mG fluorescence of platelets and megakaryocytes in blood smear, spleen and femur of mT/mG; PF4-Cre transgenic mice. (**A**,**B**) Blood smear samples of mT/mG PF4-Cre negative and positive mice were analyzed for mT and mG fluorescence of blood cells. (**A**) Representative images of mT (upper panel) and mG (lower panel) fluorescence of platelets in blood smear of transgenic mice using differential interference contrast (DIC), *n* = 8–10, scale bar 20 µm. (**B**) Representative images of single channels are shown representing either mT or mG fluorescence. Additionally, DAPI staining was performed to identify nucleus containing cells such as leukocytes in blood smear, *n* = 8–10, scale bar 10 µm. (**C**) Analysis of hematopoietic active tissue (spleen and femoral bone marrow) in mT/mG;PF4-Cre mice. Representative images of mT fluorescence (mT/mG;PF4-Cre negative) and mG fluorescence (mT/mG;PF4-Cre positive). DAPI was used to stain nuclei in spleen and femur. White arrows indicate megakaryocytes. *n* = 3.

We next investigated *mT* and *mG* labeling in the blood smear, spleen, and bone marrow of *mT/mG;PF4-Cre* mice (Figure 2). Examination of the blood smear revealed strong *mG* fluorescence of platelets as shown by fluorescence microscopy (Figure 2A,B) using blood from *PF4-Cre* positive mice. *mT* fluorescence of platelets and leukocytes was detected only in *PF4-Cre* negative mice. In contrast, DIC/brightfield images provided strong evidence that red blood cells exhibit almost no fluorescence, neither *mT* nor *mG* (Figure 2A) suggesting that the *mT/mG;PF4-Cre* reporter mouse is not appropriate for genetic labeling of red blood cells.

To test the *mT/mG* reporter mouse for lineage-specific cell appearance and morphology, we analyzed *mT* and *mG* labeling in *mT/mG;PF4-Cre* mice in different hematopoietic tissues such as spleen and bone marrow. An array of megakaryocytes can be identified by the fluorescence markers. In detail, *mT* positive megakaryocytes were identified in the bone marrow of the femur and in low numbers in the spleen of *PF4-Cre* negative mice. Accordingly, *mG* positive megakaryocytes were detected in both organs of *PF4-Cre* positive mice. Moreover, we identified a large number of platelets in the spleen that exhibit either *mT* (*PF4-Cre* negative mice) or *mG* fluorescence (*PF4-Cre* positive mice) (Figure 2C).

#### 2.1.3. Unaltered Platelet Activation and Receptor Surface Expression in *PF4-Cre* negative and *PF4-Cre* positive Mice

**Figure 3:**
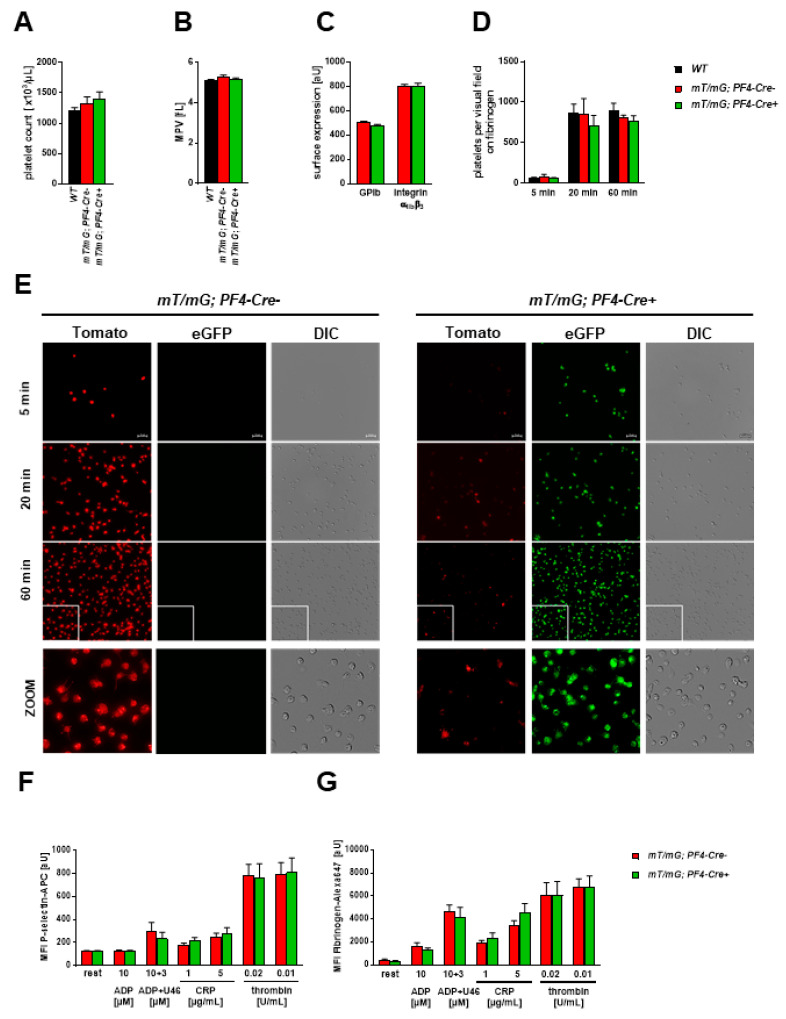
Unaltered platelet activation and receptor surface expression in *mT/mG PF4-Cre* negative and positive mice. (**A**) No differences were observed in platelet counts, (**B**) platelet size (mean platelet volume, MPV) between *WT* and *mT/mG;PF4-Cre* negative and positive mice and (**C**) in the expression of glycoprotein (GP) Ib and integrin α_IIb_β_3_ between *mT/mG;PF4-Cre* negative and positive mice. GP expression was determined using DyLight649-labeled anti-mouse GPIbα and DyLight649-labeled anti-mouse integrin α_IIb_β_3_ using flow cytometry. Statistical analysis was performed by Kruskal–Wallis test and Dunn′s post hoc test (**A**,**B**) and two-tailed Student′s t-test (**C**). Bar graphs depict mean values ± SEM, *n* = 6–8. (**D**,**E**) Isolated platelets were allowed to adhere to fibrinogen [100 µg/mL] for indicated time points and fixed with PFA. Statistical analysis was performed by two-Way ANOVA Tukey′s post hoc test. Bar graphs depict mean values ± SEM, *n* = 3–4. (**D**) The number of adherent platelets per visual field was determined. (**E**) Representative images showing platelet adhesion and platelet morphology of *mT* and *mG* fluorescent platelets, *n* = 3–4, scale bar 10 µm. (**F**,**G**) Platelet degranulation (P-selectin exposure) and fibrinogen binding (marker for integrin activation) following activation with indicated agonists was determined in washed whole blood using flow cytometry. (**F**) P-selectin externalization was analyzed with an anti-human/mouse CD62P-APC antibody, *n* = 6–7. (**G**) Fibrinogen binding was determined using fibrinogen-Alexa Fluor™ 647, *n* = 5–6. Statistical analysis was performed by two-tailed Student′s *t*-test. Bar graphs depict mean values ± SEM. CRP = collagen-related peptide, U46 = Thromboxane A2 analogue, ADP = adenosine diphosphate, DIC = differential interference contrast.

To test if platelet numbers and characteristics differ in *mT/mG;PF4-Cre* positive and negative mice, we determined different platelet parameters. First, we analyzed platelet counts and size and the exposure of platelet glycoproteins in these mice and found no significant differences (Figure 3A,C). To investigate platelets from different genotypes in more detail, we investigated platelet adhesion on fibrinogen at different time points and determined platelet activation. First, platelet adhesion to immobilized fibrinogen after 5, 20, and 60 min revealed no differences between genetically different experimental groups (Figure 3D,E). However, we detected individual platelets with *mT* fluorescence in *mT/mG;PF4-Cre* positive mice (Figure 3E, lower panel) suggesting the existence of single wildtype platelets in these mice.

To analyze platelet activation, P-selectin exposure as marker for degranulation and fibrinogen binding to integrin α_IIb_β_3_ (fibrinogen receptor) as marker for integrin activation was determined by flow cytometry (Figure 3F,G). The activation of platelets with different agonists that induce signal transduction via G-protein coupled receptors or collagen receptor activation showed no major differences between platelets isolated from *mT/mG;PF4-Cre* positive and negative mice (Figure 3F,G). Taken together, no major differences in platelet characteristics and platelet activation were found between different groups and non-recombined platelets can be identified by *mT* fluorescence suggesting no Cre recombinase activity in the respective precursor cell.

#### 2.1.4. Comparable Thrombus Formation in *PF4-Cre* negative and positive *mT/mG* transgenic Mice

**Figure 4:**
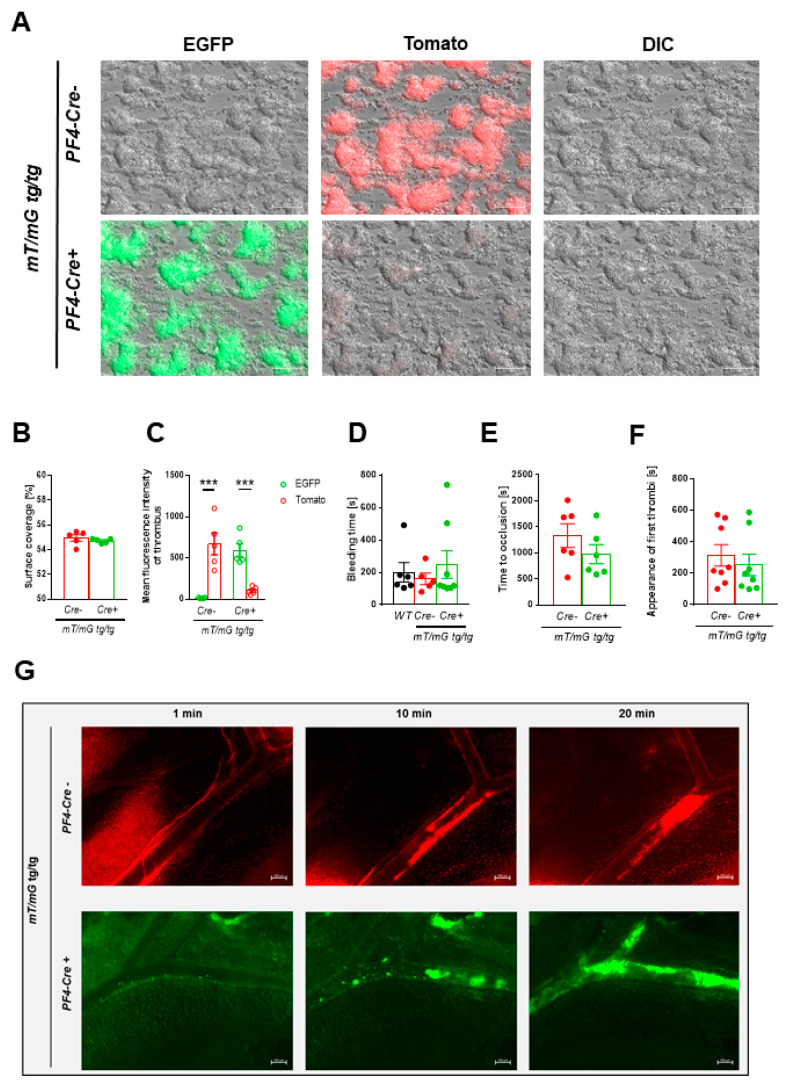
Unaltered thrombus formation in *mT/mG;PF4-Cre* negative and positive mice ex vivo and in vivo. (**A**–**C**) Murine whole blood was perfused over a collagen-coated matrix (200 µg/mL) with a shear rate of 1000 s^−1^. (**A**) Representative images of thrombus formation showing *mT* (upper panel) or *mG* (lower panel) fluorescence of platelets that have been incorporated into the growing thrombus. Determination of (**B**) surface coverage and (**C**) Mean fluorescence intensity (MFI) of thrombi to quantify thrombus formation of each genotype, *n* = 5, scale bar 50 µm. (**D**) Hemostasis was analyzed via tail bleeding time of *mT/mG;PF4-Cre* negative and positive mice. No differences between genotypes could be detected, *n* = 5–7. (**E**–**G**) In vivo thrombus formation in mesenteric arteries after FeCl_3_ induced injury was analyzed using intravital microscopy. (**E**) Time to occlusion of the injured vessel and (**F**) appearance of first thrombi were determined. (**G**) Platelet aggregate formation at indicated time point was monitored by *mT* and *mG* fluorescence of platelets. *mT* and *mG* fluorescence of platelets was stable and allowed visualization of thrombus growth until full occlusion of the vessel in *mT/mG;PF4-Cre* mice, *n* = 6–8, scale bar 100 µm. Statistical analysis was performed by two-tailed Student′s *t*-test (**B**,**C**,**E**,**F**) and Kruskal–Wallis test followed by Dunn′s post hoc test (**D**). Bar graphs depict mean values ± SEM, * *p*< 0.05, ** *p* < 0.01 and *** *p* < 0.001.

To analyze platelet activation under more physiological conditions, platelet adhesion and aggregate formation leading to three-dimensional thrombus formation under flow conditions were investigated ex vivo. To this end, cover slips were coated with 200 µg/mL collagen and whole blood from *mT/mG;PF4-Cre* positive and negative mice was perfused through the chamber at an arterial shear rate of 1000 s^−1^ (Figure 4A). As shown in Figure 4A and B, no differences in the formation of three-dimensional thrombi were observed between different groups. Whole blood from *PF4-Cre* negative mice showed strong *mT* fluorescence of platelets within the thrombus. Accordingly, the perfusion of whole blood from *PF4-Cre* positive mice resulted in strong *mG* fluorescence of the thrombus because the thrombi consist mainly of platelets and only single leukocytes and red blood cells can be found in the thrombus [20] (Figure 4A,B).

#### 2.1.5. Tracking of mT/mG positive Platelets via Intravital Microscopy 

Next, we investigated hemostasis in *mT/mG;PF4-Cre* mice. In this model, the ability of mice to arrest bleeding after cutting the tail tip with a scalpel to induce a defined tail wound serves as an indicator for normal blood clotting. In line with the ex vivo results shown above, no differences in hemostasis were observed when the time until bleeding stops was determined (Figure 4C).

To analyze the formation of arterial thrombi in vivo, *mT/mG;PF4-Cre* mice were investigated by intravital microscopy (Figure 4D–F). We injured mesenteric arterioles by topical application of FeCl_3_ and tracked *mT* and *mG* fluorescent platelets in vivo. As shown in Figure 4D–F, platelets showed bright fluorescence of either *mT* in *mT/mG;PF4-Cre* negative mice or *mG* in *mT/mG;PF4-Cre* positive mice. Furthermore, no differences were observed when we determined the appearance of first thrombi at the site of injury and the time to full occlusion of the vessel in these mice suggesting no major differences in arterial thrombosis in vivo (Figure 4D–F). 

Taken together, platelet aggregates and thrombus formation can be tracked by intravital microscopy and reveals unaltered platelet adhesion and thrombus formation in the double fluorescent cre reporter mouse.

#### 2.1.6. Transfer of Bone Marrow from *mT/mG;PF4-Cre* positive Mice to *C57BL/6* Mice 

**Figure 5:**
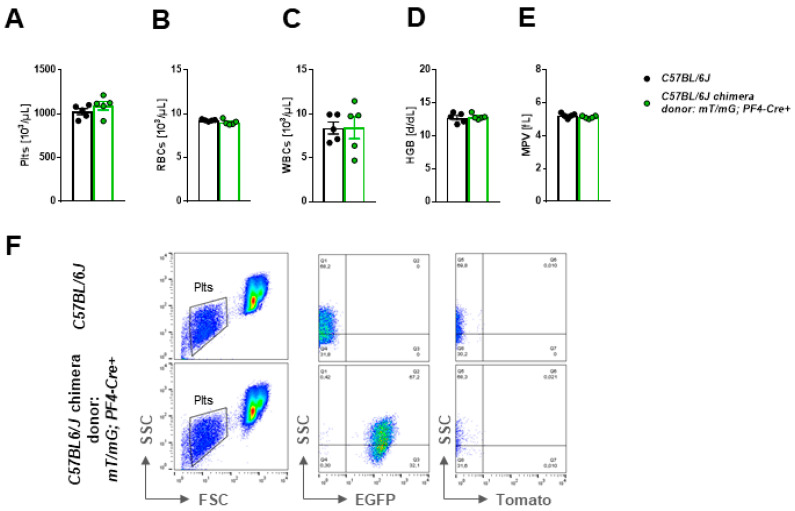
Transfer of bone marrow from *mT/mG; PF4-Cre* positive mice to *C57BL/6* mice leads to the appearance of *mG*-positive platelets in the circulation of chimeric mice. *C57BL/6* mice were exposed to sublethal irradiation and received bone marrow cells of *mT/mG;PF4-Cre* positive mice. Platelet count (**A**), number of red blood cells (**B**) and leukocytes (**C**), hemoglobin concentration (**D**) and platelet size (**E**, mean platelet volume) were determined in heparinized whole blood by a hematology analyzer (Sysmex, Norderstedt, Germany). No major differences between bone marrow chimeric mice and *C57BL/6* controls could be detected, *n* = 5. (**F**) Platelets from bone marrow chimeric mice were analyzed regarding their *mT* and *mG* fluorescence in the SSC and FSC specific profile. Only platelets with *mG* fluorescence could be detected, *n* = 5. Statistical analysis was performed by two-tailed Student′s t-test. Bar graphs depict mean values ± SEM. Plts = platelets.

The first characterization of *mT/mG* mice suggested that these mice can be used for transplantation studies [21]. Therefore, we wanted to know if the transfer of bone marrow from *mT/mG;PF4-Cre* positive mice to *C57BL/6* mice provides the advantage for platelet tracing and the analysis of platelet morphology in bone marrow chimeric mice. Bone marrow of *mT/mG;PF4-Cre* positive mice was extracted and transferred into *C57BL/6* mice. Successful transfer of bone marrow was monitored by determination of platelet counts (Figure 5A), the number of red blood cells (Figure 5B) and leukocytes (Figure 5C), hemoglobin plasma levels (Figure 5D), and mean platelet volume (platelet size) (Figure 5E). When *C57BL/6* mice were reconstituted with *mT/mG;PF4-Cre* bone marrow we were able to detect *mG* positive platelets in these mice but not in control *C57BL/6* mice using flow cytometry (Figure 5F). In summary, the transfer of bone marrow from *mT/mG;PF4-Cre* positive mice to *C57BL/6* mice led to the appearance of *mG*-positive platelets in the circulation of chimeric mice.

## 3. Discussion

The present study revealed that the double fluorescent Cre reporter mouse that expresses dtTomato prior to excision and EGFP following excision is a useful tool for tracing megakaryocytes and platelets and visualizing thrombus formation in real time by intravital microscopy. Platelets and megakaryocytes from *mT/mG;PF4-Cre* positive mice showed bright green fluorescence in the circulation (platelets) and in tissue such as the spleen (platelets and megakaryocytes) or bone marrow (megakaryocytes). Since there is no complete knockout efficiency in conditional knockout mice, the *mT/mG;PF4-Cre* mouse represents an important tool for using the Cre/*loxP* system in mice to distinguish between wildtype and knock-out platelets because platelets with *mT* fluorescence can be identified in this mouse line representing non-recombined platelets without Cre recombinase activity in the progenitor cell. No differences in platelet activation and thrombus formation have been observed between *mT*/*mG*;*PF4-Cre* positive and negative platelets and mice. Furthermore, we show that platelet count and size, platelet adhesion and bleeding times of *mT/mG;PF4-Cre* transgenic mice are comparable to wildtype controls or already published data from our group (flow chamber, injury of mesenteric arterioles) [20,21]. Thus, mating these mice with a conditional knockout mouse where the gene knockout is only in Cre-expressing cells ensures that the observed phenotype in these mice can be dedicated to the loss of a specific gene in megakaryocytes. Furthermore, the *mT/mG;PF4-Cre* mouse can be used for transplantation studies because the transplantation of bone marrow from *mT/mG;PF4-Cre* positive mice to *C57BL/6* mice allowed tracing of platelets in the circulation of bone marrow chimera.

The double fluorescent *mT/mG* mouse line provides several advantages for the analysis of platelet activation and thrombus formation. First, *PF4-Cre* genotyping of these mice is simple in an *mT/mG* homozygous background because platelets can be easily detected by flow cytometry using either the EGFP (*mG* fluorescence) or the Tomato channel (*mT* fluorescence) to distinguish between *PF4-Cre* positive and negative mice. Furthermore, the reporter mouse allows real-time visualization of platelets of both recombined and non-recombined cells at single cell resolution (Figure 4F) in vivo. The bleed-through between the fluorescent channels is minimal as already observed by Muzumdar and colleagues and both, *mT* and *mG* are photostable [22]. The absolute brightness and photostability of *mG* and *mT* fluorescence was also observed in *mT/mG;PF4-Cre* positive and negative mice allowing the tracing of platelets after vascular injury of mesenteric arterioles (Figure 4D–F).

The results of the present study clearly shows bright fluorescence of platelets either with *mT* (*PF4-Cre* negative mice) or with *mG* (*PF4-Cre* positive mice) in vitro and in vivo. However, flow cytometric analysis revealed almost no fluorescence of red blood cells, neither in *PF4-Cre* negative nor in *PF4-Cre* positive mice. This might be due to the fact, that the major difficulty of fluorescent imaging of proteins, cells, and tissues within whole animals is the light absorption by hemoglobin, as well as light scattering [23,24]. Both, absorption and scattering become less distinct as the light wavelength increases. The “optical window”, which is most transparent for the visualization in living tissues, is considered to be between 650–700 and 1100 nm suggesting the development of bright far-red or near infrared fluorescent proteins to increase sensitivity of whole body imaging techniques. However, the wavelength for tdTomato is 554/581 nm (excitation/emission) and for EGFP 488/509 nm (excitation/emission). This might explain the almost absent fluorescence of red blood cells.

Furthermore, we detected a small proportion of *mG* positive leukocytes (Figure 1C–E) raising concerns about the specificity of the *PF4* promoter for megakaryocyte/platelet-restricted Cre expression. Different groups in the past already doubted about the specificity of the *PF4* promoter [25,26] and described a broader expression of the mouse *PF4*-Cre transgene beyond the megakaryocyte lineage. Calaminus and colleagues demonstrated that the activity of the *PF4-Cre* recombinase is not restricted to megakaryocytes and platelets but extends to other myeloid and lymphoid lineages at significant levels between 15 and 60% [25]. They show that *PF4-Cre* also recombines in both fetal liver and bone marrow hematopoietic stem cells (HSCs), including the most primitive fraction containing the long-term repopulating HSCs.

In line with *mG* fluorescence of CD45 positive cells detected by flow cytometry, Pertuy and colleagues found a minor fraction of CD45-positive cells in circulating blood but also recombined cells of monocyte/macrophage origin in all tissues [26]. In contrast, a study by Rudolph and colleagues confirmed lineage specificity of *PF4-Cre* mice that have been crossed with conditional ADAP (adhesion and degranulation-promoting adapter protein) knockout mice [27]. They generated a reporter strain by crossing *PF4-Cre* mice to *Rosa26-tdRFP* (tdRFP, tandem-dimer red fluorescent protein) reporter mice and could not detect any effects of Cre recombinase in other cells beyond platelets and megakaryocytes [27]. Taken together, the use of *PF4-Cre* mice and the identification of *mG* positive cells always should be examined carefully, especially under inflammatory conditions when recombination in immune cells is further increased.

The *mT/mG Cre* reporter mouse expresses a membrane-targeted fluorescent protein. The aim to attach a membrane tag to the reporter protein was to aid in visualization of fine processes as observed in neurons [22]. Muzumdar and colleagues showed that membrane localization of a fluorescent marker is highly effective and probably superior to cytoplasmic localization to outline, e.g., cell morphology. The analysis of *mT/mG;PF4-Cre* mice revealed more cytoplasmic fluorescence of both, *mT* and *mG*, in platelets (Figure 3E) that is highly efficient to label and trace platelets but membrane localization of *mT* in leukocytes (Figure 2B). However, megakaryocytes of the bone marrow and spleen exhibit more membrane-localized fluorescence of *mT* and *mG* (Figure 2C) as already observed by Pertuy and colleagues [26] This might be due to the fact that platelets are anuclear small cells of megakaryocyte origin. Nevertheless, bright fluorescence and thus identification of platelets in *mT/mG;PF4-Cre* positive and negative mice could be observed (Figure 4F) that allows tracing of platelets not only ex vivo but also in vivo to analyze arterial thrombosis without additional labeling of platelets by a fluorescent dye (Figure 4).

Taken together, the presented results indicate that the *mT/mG;PF4-Cre* mouse line is an elegant tool for easy genotyping of mice, direct and real-time visualization of platelets in vitro and in vivo and the identification of non-recombined platelets. Thus, the double fluorescent *PF4-Cre* mice are important for the analysis of platelet-specific knockout mice to dedicate the observed phenotype of transgenic mice to the megakaryocyte/platelet lineage. However, lineage-specific tracing in these mice is leaky to a small extend because a small proportion (~13%) of CD45-positive cells exhibit *mG* fluorescence. Therefore, the analysis of platelets and their role in different cellular processes or diseases beyond hemostasis and thrombosis has been critically performed, especially when inflammation plays a role and therefore recombination in immune cells is increased.

## 4. Materials and Methods

### 4.1. Animals

Reporter mice (*Gt(ROSA)26Sor^tm4(ACTB-tdTomato,-EGFP)Luo^/J*, stock no. 007576) were purchased from The Jackson Laboratory (Bar Harbor, Maine, USA). This double fluorescent Cre reporter mouse called *mT/mG* reporter mouse expresses membrane-targeted tdTomato (′′*mT*′′) prior to Cre excision and membrane-targeted EGFP (′′*mG*′′) following Cre excision [22]. These mice were crossbred with *PF4-Cre* transgenic mice (The Jackson Laboratory, stock no. 008535, *C57BL/6-Tg(Pf4-icre)Q3Rsko/J,* Bar Harbor, Maine, USA) to confine the expression of EGFP to megakaryocytes and platelets [19]. The animals were bred in the animal facility of University Düsseldorf. As *WT* controls served either littermates, which had a wildtype genotype for *mT/mG* and *C57BL/6* mice, which were bought at Janvier Labs (Le Genest-Saint-Isle, France).

All animal experiments were performed under adherence of the EU Directive 2010/63/EU and German animal welfare act using a protocol approved by the Heinrich-Heine-University Animal Care Committee and he district government of North Rhine-Westphalia (LANUV NRW, permit numbers: 84-02.05.20.12.284, 84-02.05.40.16.073, 84-02.04.2013.A210 and 84 02.04.2016.A493).

### 4.2. Chemicals

Platelets were activated with ADP (Sigma-Aldrich, St. Louis, MO, USA), thrombin (Roche, Basel, Switzerland), CRP (Richard Farndale, University of Cambridge, United Kingdom) and U46619 (Enzo Life Sciences, Lörrach, Germany). Human fibrinogen (Sigma-Aldrich, St. Louis, MO, USA), collagen (Takeda Pharma, Berlin, Germany), heparin (Ratiopharm, Ulm, Germany), prostacyclin (Calbiochem, Sigma-Aldrich, St. Louis, MO, USA)), and apyrase (Sigma-Aldrich, St. Louis, MO, USA) were purchased.

### 4.3. Murine Platelet Preparation and Determination of Cell Count

Murine platelet preparation followed a previously described protocol [28,29]. Blood was drawn from anaesthetized mice via retro-orbital plexus and collected in 300 μL heparin (20 U/mL). The blood was centrifuged at 250 g at 22 °C for 5 min. The resulting supernatant was centrifuged at 50 g for 6 min to obtain platelet-rich plasma (PRP). PRP was centrifuged at 650 g using apyrase and PGI_2_ for 5 min. The remaining pellet was resuspended in Tyrode′s buffer (136 mM NaCl, 0.4 mM Na_2_HPO_4_, 2.7 mM KCl, 12 mM NaHCO_3_, 0.1% glucose, 0.35% bovine serum albumin (BSA, Sigma-Aldrich, St. Louis, MO, USA), pH 7.35, apyrase (0.02 U/mL) and prostacyclin (0.5 µM)) and centrifuged at 650 g for 5 min. Depending on the following experiment platelets were either resuspended in Tyrode′s buffer (pH 7.35) with or without CaCl_2_ (2 mM). Platelets counts and MPV were analyzed by a hematology analyzer (Sysmex, Norderstedt, Germany).

### 4.4. Flow Cytometry

Flow cytometry analysis was performed as described elsewhere [5]. An analysis of murine platelet activation was performed using fluorophore-labeled antibodies for P-selectin expression (Anti-Human/Mouse CD62P APC, Clone Psel.K02.3, eBioscience, San Diego, California, USA, final concentration 20 µg/mL) and fibrinogen binding (Fibrinogen From Human Plasma, Alexa Fluor™ 647 Conjugate, Life Technologies, Carlsbad, California, USA, final concentration 50 µg/mL). Heparinized blood was diluted in 500 µL Tyrode′s buffer (pH 7.35) and washed twice via centrifugation at 650 at 22 °C for 5 min. The remaining pellet was resuspended in 500 μL Tyrode′s buffer (pH 7.35) supplemented with CaCl_2_ (2 mM). Platelets were stimulated with ADP (10 µM), ADP and U46619 (10 µM and 3 µM), CRP (1 and 5 µg/mL) and thrombin (0.02 and 0.01 U/mL) at 22 °C for 15 min. The reaction was stopped by the addition of PBS (Sigma-Aldrich, St. Louis, MO, USA) and samples were analyzed by use of a FACSCalibur flow cytometer (BD Biosciences, Heidelberg, Germany).

To analyze surface expression of different glycoproteins, blood samples were mixed with the antibodies DyLight649-labeled rat anti-mouse/human integrin α_IIb_β_3_ (GPIIIa, CD61) (emfret Analytics, Würzburg, Germany, M025-3, no stock concentration provided by emfret Analytics, used dilution 1:10) and DyLight649-labeled anti-mouse GPIbα (Emfret Analytics, Würzburg, Germany, no stock concentration provided by emfret Analytics, used dilution 1:10), and incubated at 22 °C for 15 min before measurements.

To label the different blood cell types washed whole blood was incubated with APC-labeled rat anti-mouse TER-119 (BD Bioscience, Heidelberg, Germany, final concentration 20 µg/mL), DyLight649-labeled anti-mouse GPIbα (Emfret Analytics, Würzburg, Germany, no stock concentration provided by Emfret Analytics, used dilution 1:10) and APC-labeled rat anti-mouse CD45 (BD, Heidelberg, Germany, final concentration 20 µg/mL) and analyzed using the Tomato (*mT* fluorescence) and the EGFP channel (for *mG* fluorescence).

### 4.5. Fluorescence Microscopy

For visualization, two drops of peripheral blood were added to 50 µL heparin (20 U/mL). Then one drop was spread on a cover slip and the resulting blood smears were stained with DAPI (Roche Diagnostics, Mannheim, Germany final concentration 1.6 µg/mL) to stain nuclei of leukocytes. Images were taken with the Zeiss LSM 510-META Confocal laser scanning microscope (Carl Zeiss, Oberkochem, Germany).

Sample slices were prepared from paraffin embedded spleen and femur samples, dewaxed and dehydrated, stained with DAPI (Roche Diagnostics, Mannheim, Germany, final concentration 1.6 µg/mL) and inundated with FluoromountTM (Sigma-Aldrich, St. Louis, MO, USA). The documentation was done after drying at 4 °C overnight with microscope Axio Observer.D1 (Carl Zeiss Microscopy GmbH, Oberkochem, Germany, AxioCam MRm, objective LD Plan-Neofluar 40x 0.6 Korr Ph2 M27) and the resulting pictures were analyzed using ImageJ-win64 (ImageJ 1.52, freeware by National Institutes of Health, USA) and ZEN 2.6 (Zen 2.6 blue edition, Carl Zeiss Microscopy GmbH, Oberkochem, Germany).

### 4.6. Flow Chamber Experiments to Analyze Thrombus Formation under Flow Conditions

Coverslips (24 × 60 mm) were coated with 200 μg/mL fibrillar type I collagen (Takeda Pharma, Berlin, Germany) overnight and then blocked with 1% BSA solution for at least 60 min. Whole blood samples were perfused through the flow chamber at a shear rate of 1000 s^−1^ and platelet adhesion and thrombus formation were recorded using the Microscope Axio Observer.D1 (Carl Zeiss Microscopy GmbH, Oberkochem, Germany, AxioCam MRm, objective LD Plan-Neofluar 40x 0.6 Korr Ph2 M27). The images were analyzed for three-dimensional thrombus formation using ZEN 2.6 (blue edition, Carl Zeiss Microscopy GmbH, Oberkochem, Germany).

### 4.7. Platelet Adhesion and Spreading

Experiments were performed as described previously [21,29]. Coverslips (24 × 60 mm) were either coated with 100 µg/mL fibrinogen or 200 μg/mL type I collagen at a defined area (10 × 10 mm) and incubated overnight at 4 °C. Afterwards the coverslips were blocked with 1% BSA for 60 min. Then, 2 × 10^4^ isolated platelets were resuspended in 70 μL Tyrode′s buffer (pH 7.35) supplemented with CaCl_2_ (2 mM), applicated to the prepared coverslips and incubated at room temperature for indicated time points. Non-adherent platelets were carefully removed by rinsing 2× with PBS. The preparation was fixed by 4% phosphate buffered formaldehyde at 4 °C for 10 min, subsequently rinsed again carefully with PBS and inundated with FluoromountTM (Sigma-Aldrich, St. Louis, MO, USA). Platelet adhesion was documented after drying at 4 °C overnight using the microscope Axio Observer.D1 (Carl Zeiss Microscopy GmbH, Oberkochem, Germany, AxioCam MRm, objective LD Plan-Neofluar 40x/0.6 Korr Ph2 M27). The images were analyzed ImageJ-win64 (ImageJ 1.52, freeware by National Institutes of Health, USA) and ZEN 2.6 (Zen 2.6 blue edition, Carl Zeiss Microscopy GmbH, Oberkochem, Germany).

### 4.8. Intravital Microscopy of Arterial Thrombosis in Mesenteric Arterioles Following Injury with FeCl_3_

Mice (4–5 week of age) were anesthetized with Ketamin (Ketaset, Zoetis, Parsippany-Troy Hills Township, New Jersey, USA, 100 *mG*/kg) and Xylazin (WDT, Garbsen, Germany, 5 *mG*/kg) by intraperitoneal (i.p.) injection. After a midline abdominal incision, the mesentery was exteriorized and arterioles free of fat tissue were injured by topical application of a filter paper saturated with 20% FeCl_3_ (Sigma-Aldrich, St. Louis, MO, USA) for 20 s. Thrombus formation was observed with a fluorescence microscope (Axio Observer.D1 Carl Zeiss Microscopy GmbH, Oberkochem, Germany, AxioCam MRm, objective APlan 10x/0.25 Ph1). Time until full occlusion of the vessel (when blood flow had stopped for more than 60 s) was measured. Experiments were stopped after 40 min when no occlusion of the injured vessel was detected.

### 4.9. Determination of the Bleeding Time to Investigate Hemostasis in Transgenic Mice

Mice at the age of 10–12 weeks were anesthetized with Ketamin (100 *mG*/kg) and Xylazin (5 *mG*/kg) i.p. and placed in prone position. A transverse incision was made with a scalpel at a position where the diameter of the tail is 2.25–2.5 mm using a gauge to cut a defined size of the tail tip. The tail was immediately immersed in a 15 mL Falcon tube containing pre-warmed PBS in a water bath to 37 °C and bleeding was continuously monitored. Each animal was monitored for 20 min even if bleeding stopped in order to detect any re-bleeding events. The time from the incision to the cessation of bleeding was recorded using a stop clock.

### 4.10. Generation of Bone Marrow Chimeric Mice

Twelve-week-old male *C57BL/6J* mice were exposed to sublethal irradiation of 10 Gray for 3.32 min using the gamma-irradiation device Biobeam GM 2000 (Gamma Service Medical, Leipzig, Germany). Donor bone marrow cells were isolated from femur and tibia of *mT/mG;PF4-Cre* positive mice. After cervical dislocation of the donor animals, the femurs and tibias were freed from the tissue and released from the hip anchorage. Then both hind leg bones were cut off at the distal and proximal ends just behind the joints. Following, the bone marrow cavity was rinsed with chilled PBS through a nylon filter into a reaction tube using a 26 G cannula (Braun, Melsungen, Germany) and centrifuged at 500 g for 10 min. Lysis of erythrocytes with 1 mL erythrocyte lysis buffer (155 mM NH_4_Cl, 10 mM KHCO_3_, 0.1 mM EDTA; pH = 7.35) for 5 min on ice followed. The suspension was centrifuged again at 500 g for 10 min, the supernatant was removed, and the pellet was resuspended in 150 µL PBS and kept on ice until application into recipient animals via the retro-bulbar venous plexus. Bone marrow from one donor hind leg was used per recipient mouse with a minimum of 5 × 10^6^ bone marrow cells. The application volume was set at 150 µL of cell suspension. The recipient mice that have been irradiated had the same genotype and sex as the donor mice and were of similar age. After the application of the donor bone marrow, mice were kept under sterile conditions for approximately 2 weeks and supplemented with neomycin through drinking water according to the manufacturer′s instructions (Sigma-Aldrich, St. Louis, MO, USA). After a convalescence period of 8 weeks, blood cell counts were determined, and blood cells were analyzed using BD FACSCalibur.

### 4.11. Statistical Analysis

Data are shown as mean ± standard error of mean (SEM). Statistical significance was analyzed using GraphPad Prism 7 software (San Diego, Californa, USA) and the recommended tests were used as indicated. *p* values <0.05 were considered to be statistically significant.

## 5. Conclusions

The megakaryocyte/platelet specific double fluorescent Cre reporter mouse *mT/mG;PF4-Cre* represents an elegant model for lineage tracing, the analysis of cell morphology, transplantation studies, and the differentiation of recombined versus non-recombined platelets. However, a minor fraction of leukocytes showed *mG* fluorescence in *mT/mG;PF4-Cre* positive mice. Thus, the role of platelets in inflammatory diseases should be assessed carefully and *mG* fluorescent cells should be examined for their origin.

## Abbreviations

ADP	adenosine diphosphophate
BSA	bovine serum albumine
CaCl_2_	calcium chloride
CLEC-2	C-type lectin-like receptor 2
Cre	cyclization recombination or causes recombination
CRP	collagen-related peptide
DIC	differential interference contrast
EDTA	ethylenediaminetetraacetic acid
FeCl_3_	iron(III) chloride
GPIb/VI	glycoprotein (GP) Ib / VI
HE	hematoxylin/eosin
KCl	potassium chloride
KHCO_3_	potassium bicarbonate
loxP	locus of X-over P1, recognition site for cre to excision
MFI	mean fluorescence intensity
mG	membrane-targeted enhanced green fluorescent protein (EGFP)
min	minute
mT	membrane-targeted tandem dimer Tomato (red fluorescent protein)
NaCl	sodium chloride
NaHCO_3_	sodium bicarbonate
Na_2_HPO_4_	disodium phosphate
NH_4_Cl	ammonium chloride
PLD1	phospholipase D1
PF4	platelet factor 4
PFA	paraformaldehyde
PRP	platelet-rich plasma
rest	resting
Thr	thrombin
U46	U46619, thromboxane analogue
vWF	von Willebrand Factor
